# A Novel Thermostable and Alkaline Protease Produced from *Bacillus stearothermophilus* Isolated from Olive Oil Mill Sols Suitable to Industrial Biotechnology

**DOI:** 10.3390/molecules26041139

**Published:** 2021-02-20

**Authors:** Aida Karray, Mona Alonazi, Habib Horchani, Abir Ben Bacha

**Affiliations:** 1 Laboratoire de Biochimie et de Génie Enzymatique des Lipases, ENIS Route de Soukra, Université de Sfax-Tunisia, Sfax 3038, Tunisia; 2 Biochemistry Department, Science College, King Saud University, P.O. Box 22452, Riyadh 11495, Saudi Arabia; moalonazi@ksu.edu.sa; 3 Groupe de Recherche en Environnement et Biotechnologie, Science Department, College of Rivière-Du-Loup, Rivière-Du-Loup, Québec, QC G5R 1R1, Canada; habib.horchani@cegeprdl.ca; 4 Laboratory of Plant Biotechnology Applied to Crop Improvement, Faculty of Science of Sfax, University of Sfax, Sfax 3038, Tunisia

**Keywords:** protease, *Bacillus stearothermophilus*, characterization, thermostable

## Abstract

This study was conducted to identify a new alkaline and thermophilic protease (Ba.St.Pr) produced from *Bacillus stearothermophilus* isolated from olive oil mill sols and to evaluate its culture conditions, including temperature, pH, carbon and nitrogen sources, and incubation time. The optimum culture conditions for cell growth (10 g/L) and protease production (5050 U/mL) were as follows: temperature 55 °C, pH 10, inoculation density 8 × 10^8^ CFU/mL, and incubation time 24 h. The use of 3% yeast extract as the nitrogen sources and galactose (7.5 g/L) as the carbon sources enhanced both cell growth and protease production. Using reversed-phase analytical HPLC on C-8 column, the new protease was purified with a molecular mass of approximately 28 kDa. The N-terminal sequence of Ba.St.Pr exhibited a high level of identity of approximately 95% with those of *Bacillus* strains. Characterization under extreme conditions revealed a novel thermostable and alkaline protease with a half-life time of 187 min when incubated with combined Ca^2+^/mannitol. Ba.St.Pr demonstrated a higher stability in the presence of surfactant, solvent, and Ca^2+^ ions. Consequently, all the evaluated activity parameters highlighted the promising properties of this bacterium for industrial and biotechnological applications.

## 1. Introduction

Proteases are proteolytic enzymes that decompose proteins by hydrolyzing their peptide bonds. Although proteases are derived from different sources such as animals, plants, and microorganisms, microbial enzymes are characterized with simple and rapid production, independently of climatic conditions or seasonal changes [1,2,3]. In particular, production of extracellular proteases by microorganisms is more interesting than their intracellular production because this process simplifies the downstream processing, thereby further lowering the costs [4].

In the literature, several microorganisms are described as protease producers, such as *Aspergillus oryzae* [5], *Serratia marcescens* [6], and *Bacillus subtilis* [7]. The genus *Bacillus* is the most prominent source among all the investigated bacteria. This finding is related to the high capacity for protein secretion with more than 20 g/L protein [8] and to the production of neutral and alkaline proteases [9,10], which is an interesting aspect for enzyme use in industries [11,12].

In fact, proteases produced by *Bacillus* possess attractive characteristics for several industrial and biotechnological applications, realizing that they represent approximately 60% of the total worldwide enzyme sales. It is well documented that the proteases produced by *Bacillus* were largely used in the detergent industry owing to their extensive pH and temperature activity and stability range [13]. In particular, the enzymes applied in this industry should be able to preserve their activity in an alkaline environment and their stability in the presence of several toxic compounds, including oxidants and surfactants. Furthermore, proteases produced by *Bacillus* are potentially used in the food industry for obtaining bioactive peptides and processing diverse foods [14,15]. Another characteristic of proteases produced by *Bacillus* is their stability in organic solvents, due to which they have been applied during organic synthesis [16].

Therefore, the special industrial interest on organisms belonging to the genus *Bacillus* is first due to their rapid growth rates, permitting short fermentation cycles, and second due to their expressive ability for secreting significant amounts of proteins into the extracellular medium. *Bacillus*-derived proteases are generally alkaline with optimum pH activity values of >7.0. The biochemical description of these proteases has been a significant issue, considering their use and biochemical kinetic parameters. Consequently, the catalytic parameters of proteases, such as thermoresistance and tolerance to a diverse range of pH values, stability and activity of the enzyme, and further extreme reaction conditions, are commonly investigated for each potential enzyme. The molecular weight of *Bacillus*-derived proteases ranges from 27 to 71 kDa, with an optimal pH range between 6 and 10 and an optimal temperature obtained between 37 °C and 60 °C. Moreover, the proteolytic enzymes exhibit significant stability values over a wide range of pH values and temperatures. Regarding their properties for use in biotechnological applications, bacterial proteases have been widely purified and characterized. As a result, their purity and homogeneity obviously allow determining the primary amino acid sequences and the 3D structures of proteases. Therefore, investigating the structure–function relationships of pure proteases can provide supplementary explanations about the kinetic parameters and characteristics of enzymatic catalyses [9]. Although there are several reports on the proteases produced by *Bacillus*, there is currently an increase in research on the identification of new strains producing preferment proteases from biological biotopes. In this study, we demonstrate the optimization of culture conditions, purification, and biochemical characterization of a novel thermostable and alkaline protease from *Bacillus stearothermophilus* isolated from olive oil mill sols and suitable to industrial biotechnology.

## 2. Results

### 2.1. Effect of Different Parameters on Cell Growth and Protease Production from B. stearothermophilus

#### 2.1.1. Effect of Temperature

**Figure 1:**
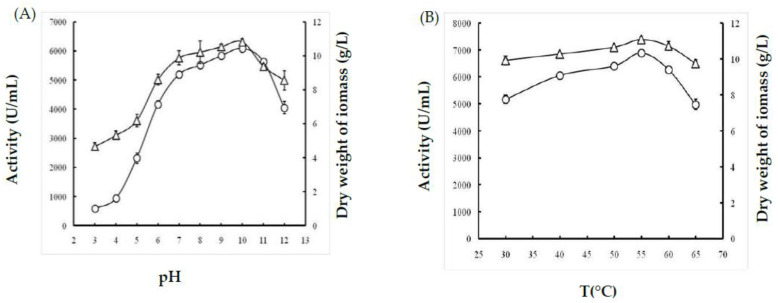
Effects of (**A**) temperature and (**B**) pH on growth (triangles) and protease production (bars) from *Bacillus stearothermophilus*. Data are expressed as mean (*n* = 3) ± SD.

Culture temperature is considered as one of the essential factors influencing both bacterial growth and their capacity to produce protease enzymes. Therefore, *Bacillus* strain was grown at temperatures ranging from 30 °C to 65 °C. In the present study, the isolated bacteria exhibited optimum cell growth at a temperature between 50 °C and 60 °C and maximum growth at 55 °C (11.05 g/L ± 0.155). Similarly, the production of protease enzymes was achieved between 50 °C and 60 °C, with a maximal activity of 6885 U/mL ± 91.923 at 55 °C (Figure 1B).

The ability of thermophilic bacteria to resist high temperatures can probably be explained by the activity in their metabolic functions associated with their physiological adaptations.

#### 2.1.2. Effect of pH

Evidently, the changes in pH values affected both cell growth and protease activity. In fact, it was observed that at acid pH values, the bacterial growth and protease productivity were extremely low (4.662 g/L ± 0.229 and 597.5 U/mL ± 24.748, respectively) compared to those obtained at pH ranging from 7 to 11. The highest dry weight of biomasses (10.82 g/L ± 0.183) and protease activity (6090 U/mL ± 155.563) were observed at pH 10 (Figure 1B). Consequently, the metabolic pathways of microorganisms were affected by pH.

#### 2.1.3. Effects of the Carbon Source

**Figure 2:**
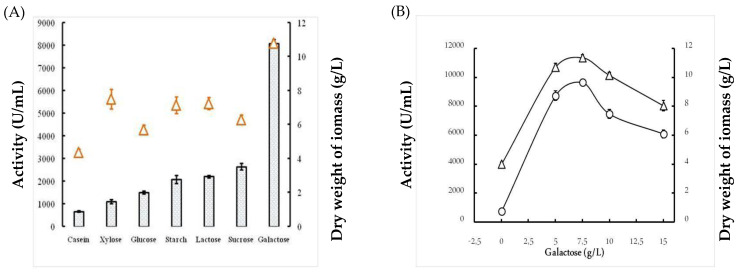
Effects of carbon source (**A**) and galactose concentration (**B**) on growth (triangles) and protease production (bars) from *Bacillus stearothermophilus*. Data are expressed as mean (*n* = 3) ± SD.

First, the effect of several carbon sources on protease activity and dry weight biomass was explored. The effects of casein, xylose, glucose, starch, lactose, glucose, and galactose were evaluated separately. Results shown in Figure 2A indicate comparable growth rates for most of the various carbon sources used in this study, except casein. Moreover, the protease activity was strongly influenced by the carbon sources. In fact, galactose permitted the highest protease activity of 8077 U/mL ± 173, followed by sucrose with a protease activity of 2647 U/mL ± 144. Next, the galactose concentration used in the culture media was optimized, with values ranging from 0 to 15 g/L. Figure 2B shows that 7.5 g/L galactose permitted the highest protease activity of approximately 9650 U/mL and the best biomass productivity of approximately 11.35 g/L. Beyond this galactose concentration, both the activity of protease and the cell growth were significantly decreased.

#### 2.1.4. Effects of Nitrogen Sources

**Figure 3:**
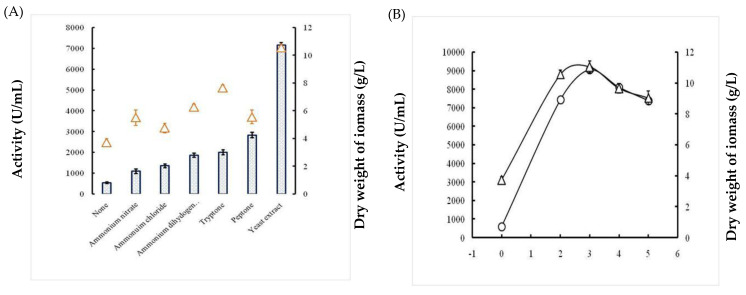
Effects of nitrogen source (**A**) and selected yeast extract concentration (**B**) on growth (triangles) and protease production (bars) from *Bacillus stearothermophilus*. Data are expressed as mean (*n* = 3) ± SD.

As described earlier, different organic and inorganic nitrogen sources (1%) were evaluated for their effects on both growth and protease activity from the examined *Bacillus*. The curves shown in Figure 3A indicate that the use of yeast extract allowed maximum cell growth (10.525 g/L ± 0.243) and protease production (7170 U/mL ± 113.13). The protease activity appeared to be significantly affected by the various nitrogen sources. In fact, microorganisms require different specific nitrogen supplements for growth and metabolism. To better ameliorate enzyme productivity and biomass growth, the selected yeast extract was used at various concentrations ranging from 0% to 5% (Figure 3B). Evidently, the use of 3% yeast extract improved both proteolytic activity and dryweight biomass, with the values reaching 9050 U/mL ± 212 and 11.05 g/L ± 0.12, respectively.

#### 2.1.5. Effects of Incubation Time and Inoculum Size

**Figure 4:**
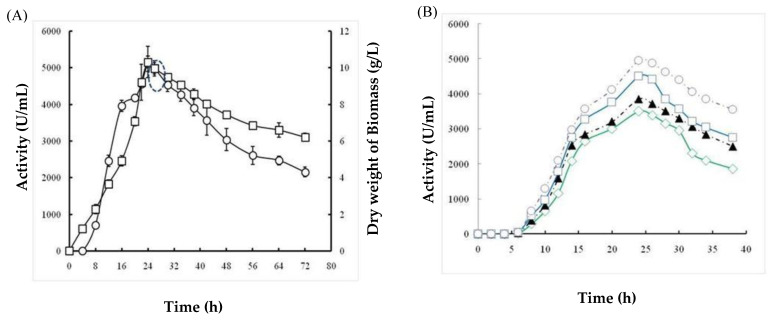
Effect of incubation time (**A**) on growth (white squares) and protease activity (white circles) and effect of inoculum size (**B**) on protease activity; (green line; 10^8^ CFU/mL) (black line; 2 × 10^8^ CFU/mL) (blue line; 4 × 10^8^ CFU/mL) (purple line; 8 × 10^8^ CFU/mL).

First, the effects of incubation time on bacterial growth and protease production were examined from 3 to 72 h and are presented in Figure 4A. The obtained curves revealed a significant increase in bacterial growth during the first few hours of bacterial culture, and the highest growth was observed at 24 h (10 g/L ± 0.8). In addition, protease production was proportional to cell growth, with an optimum value of 5100 ± 212 U/mL obtained at 24 h. Thereafter, there was a rapid decrease in protease activity and cell productivity by up to 72 h at a constant rate (Figure 4A). Next, the effect of inoculum size ranging from 10^8^ to 8 × 10^8^ CFU/mL was examined during the culture period (Figure 4B). With all the tested inoculum sizes, the protease activity increased rapidly, reaching the maximum after 24 h, and then decreased considerably. As shown in Figure 4B, the protease activity is proportional to the inoculum size tested, and the highest productivity (5050 U/mL) was observed with 8 × 10^8^ CFU/mL. Therefore, an extended inoculum size did not increase the enzyme productivity by *B. stearothermophilus.*

### 2.2. Purification Procedure of Ba.St.Pr and Determination of NH_2_-Terminal Amino Acid Sequence

**Figure 5:**
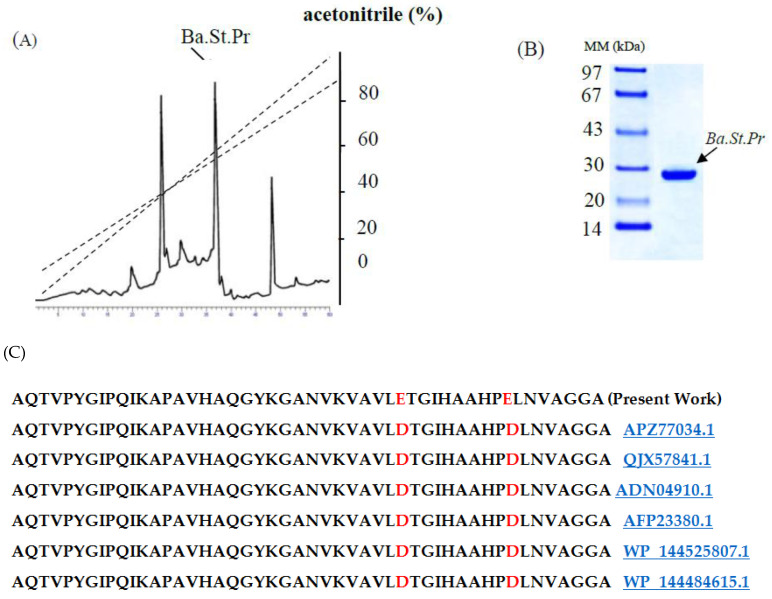
(**A**) Reversed-phase analytical HPLC on C8 matrix. Reversed-phase (RP)-HPLC eurospher 100, C-8 column (250 × 4.6 mm), was equilibrated in water. Protein elution was performed with an acetonitrile linear gradient (0–80%) at a flow rate of 1 mL/min over 60 min. 20 µL of protease (2 mg/mL). (**B**) SDS-PAGE of pure Ba.St.Pr (10 µg). (**C**) Local alignment of the Ba.St.Pr N-terminal sequence (present work) with the highest identity protease sequences from *Bacillus* strains. Identical amino acids in bold and different amino acids are in red.

The Ba.St.Pr enzyme was purified from the optimized culture media supernatant as described in the materials and methods section. Briefly, after ammonium sulfate precipitation (30–65%) and a heat treatment (80 °C for 30 min), the obtained proteins were loaded onto a reversed-phase analytical HPLC on C8 matrix equilibrated in water. The resulting protein elution profile indicated that the pure protease was eluted as a unique elution symmetrical peak at a retention time of 35 min using an acetonitrile linear gradient (0–80%) at a flow rate of 1 mL/min over 60 min (Figure 5A). The fractions exhibiting the protease activity emerged in a single peak at 35 min (Figure 5A). As summarized in Table 1, the purified Ba.St.Pr is characterized by a high specific activity of 59,022 U/mg under optimum assay conditions. Using the optimized procedure, 300 mL of culture media permitted the production of 26 mg of pure enzyme. Next, SDS-PAGE was performed to analyze the purity of Ba.St.Pr (Figure 5B). The molecular mass of the purified enzyme was found to be approximately 28 kDa. The NH_2_-terminal amino acid sequence of Ba.St.Pr allowed the clear identification of 50 residues of the pure protease as follows: AQTVPYGIPQIKAPAVHAQGYKGANVKVAVLETGIHAAHPELNVAGGA (Figure 5C). The NH_2_-tertminal sequence exhibited high identity with those reported for proteases produced by several other *Bacillus* spp., reaching 95% similarity with alkaline serine protease produced by *B. altitudinis*, alkaline protease produced by *B. pumilus,* serine alkaline protease produced by *B. circulans*, and serine protease produced by *Alkalihalobacillus lehensis*, with the accession numbers *APZ77034.1*, *QJX57841.1*, *ADN04910.1*, and *AFP23380.1*, respectively. These results strongly suggested that the Ba.St.Pr enzyme was a novel serine protease.

### 2.3. Effects of Extreme pH and Temperature on Protease Activity and Stability

**Figure 6:**
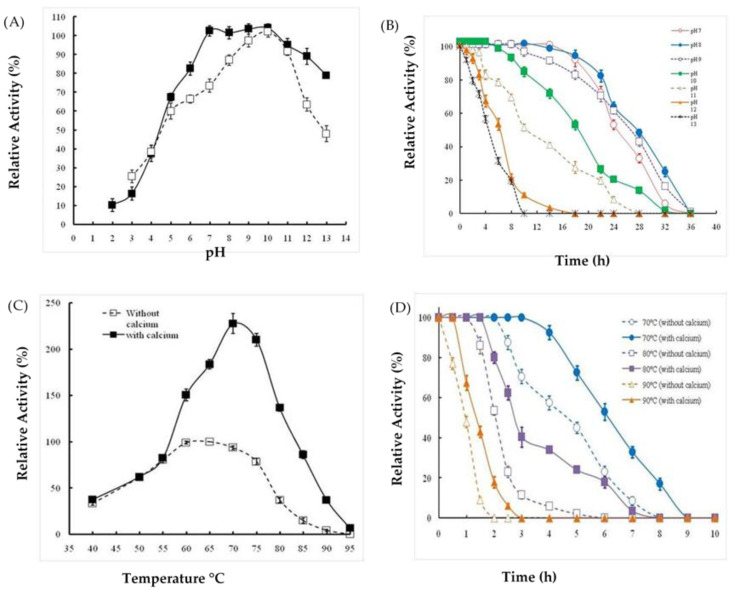
Evaluation of pH effect on the activity (white squares) and stability (black squares) of Ba.St.Pr. (**A**). Effect of alkali pH on Ba.St.Pr stability at various incubation times from 0 to 36 h (**B**). Evaluation of the effect of temperature on protease activity without Ca^2+^ ions (white squares) and with Ca^2+^ ions (black squares) of Ba.St.Pr. (**C**). Effect of extreme temperatures on Ba.St.Pr stability at various incubation times from 0 to 10 h (**D**).

Results shown in Figure 6A indicate that Ba.St.Pr exhibited high relative activity at pH values ranging from 7 to 12, with an optimum activity at pH 10. However, at pH 5 and 13, the relative activities were approximately 50%. Moreover, we interestingly noticed that the purified enzyme was strongly stable in the pH range of 7–11 after 2 h of incubation. The enzyme retained 89% and 82% of its activity at pH 6 and 12, respectively, under the same conditions. To better characterize and evaluate the pH stability, the enzyme was incubated at pH from 7 to 13 at various time periods from 0 to 36 h. At pH < 9, the enzyme retained 90% of its initial activity for an incubation time of 20 h. At pH 10, it remained stable (90%) for 10 h. Moreover, at pH > 10, the stability of Ba.St.Pr reached 80% after 4 h of incubation (Figure 6B).

Ba.St.Pr was fully active at 60 °C and 65 °C in the absence of CaCl_2_, at pH 10, and using casein as substrate. Interestingly, the protease activity was much higher when incubated with 3 mM calcium. In fact, the relative activity increased by approximately 50% and 127% at 60 °C and 70 °C, respectively (Figure 6C). Even at the highest temperature tested, Ba.St.Pr was fully active, and there was an increase in its activity by 38% at 80 °C. Moreover, to better characterize the enzyme stability at extreme temperatures, the residual activity was measured after incubations at 80 °C and 90 °C with 3 mM Ca^2+^ or with a combination of Ca^2+^ and mannitol for time intervals of 1–6 h. Evidently, as shown in Figure 6D, Ba.St.Pr is extremely stable as it retained its activity at 80 °C for 4 h combined to its activity obtained with Ca^2+^ and mannitol combination. In fact, both calcium and mannitol significantly improved the thermal stability of the enzyme as it remained active (90%) at 90 °C. The half-life time deduced from Figure 6D was approximately 123 min at 80 °C for Ba.St.Pr alone; however, it improved to 166 and 187 min when the enzyme was incubated with Ca^2+^ and the combination of Ca^2+^/mannitol, respectively.

### 2.4. Effects of Surfactants, Oxidizing Agents, Organic Solvents, and Ions on Ba.St.Pr Stability

**Figure 7:**
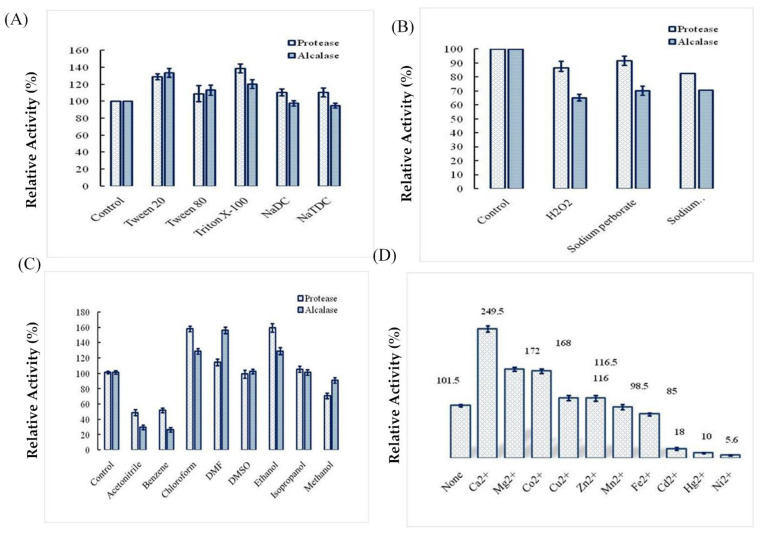
Effects of surfactants (**A**), oxidizing agents (**B**), organic solvents (**C**), and divalent ions (**D**) on Ba.St.Pr stability. The enzyme was incubated with the appropriate agent for 1 h, and the residual activity was measured at the optimal conditions. A commercial Alcalase 2.5 L, type DX, was used as a positive control.

When Ba.St.Pr was preincubated in the presence of several commercially ionic and nonionic surfactants, natural surfactants, and denaturing agents, its activity and stability increased compared with the negative control. A higher stability was observed in the presence of Triton X-100, Tween 20, or Tween 80, with the residual activities being 140%, 130%, and 120%, respectively. Ba.St.Pr retained 90%, 86%, and 82% of its activity after treatment with sodium perborate, H_2_O_2_, and sodium hypochlorite, respectively. However, Alcalase 2.5 L, type DX, retained 60% of its initial activity after a similar treatment (Figure 7A,B). Moreover, the stability of proteases in organic solvents is an important feature to discuss about its application during organic synthesis. Hence, several solvents were tested for protease stability. As shown in Figure 7C, the enzyme Ba.St.Pr was remarkably stable in chloroform and ethanol as it expressed 160% of its activity, followed by that in DMF, isopropanol, and DMSO, with the residual activity being 100%. For further comparison, the stability of Ba.St.Pr was found to be almost higher than that of commercial Alcalase 2.5 L, type DX. Several divalent metallic ions were evaluated for their effects on Ba.St.Pr activity (Figure 7D). There was a remarkable improvement in its activity by 149%, 72%, and 68% with CaCl_2_, MgCl_2_, and CoCl_2_, at 3 mM concentration, compared with the control, respectively. Moreover, the enzyme was slightly activated by Cu^2+^, Mn^2+^, and Zn^2+^ but completely inactivated by Ni^2+^, Hg^2+^, and Co^2+^.

### 2.5. Stability and Compatibility of Ba.St.Pr with Solid and Liquid Commercial Laundry Detergents

**Figure 8:**
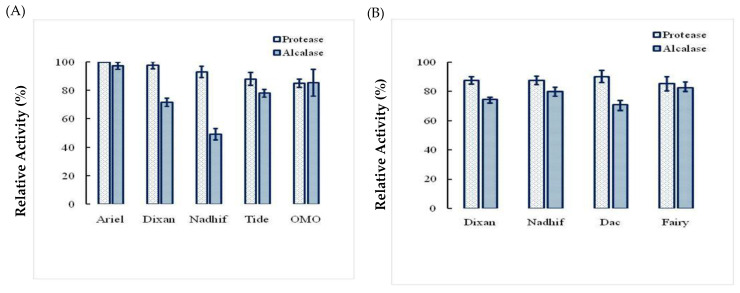
Stability and compatibility of Ba.St.Pr with solid (**A**) and liquid (**B**) commercial laundry detergents compared with commercial Alcalase 2.5 L measured under the same conditions.

The data shown in Figure 8A,B indicate that compared with Alcalase 2.5 L, type DX, Ba.St.Pr is almost extremely stable and compatible with the commercial liquid and solid laundry detergents used in this study. In fact, it retained 100% of its initial activity with several solid detergents and 90% with liquid detergents. However, the residual activities of Alcalase 2.5 L, type DX, were 70% and 60% with solid and liquid detergents, respectively.

## 3. Discussion

The use of thermostable enzymes in several industrial processes or biotechnological applications has several virtues compared with mesophilic enzymes. In fact, thermoenzymes are more resistant to thermal and chemical denaturations than mesoenzymes. These properties allow their usage in extreme conditions wherein their mesophilic equivalents would be less efficient. The thermostability properties of thermophilic enzymes have attracted the interest of several industrial sectors. In fact, thermoenzymes are generally used in the pulp and paper, food, pharmaceutical, detergents, and organic synthesis industries. Proteases, cellulases, amylases, and lipases are among the most sought-after thermophilic enzymes in these sectors of activity.

Moreover, several *Bacillus* strains are capable of producing a large amount of industrially interesting alkaline and neutral proteases [10,17].

The optimization of *Bacillus* bacterial protease production includes several parameters and starts by determining the effect of temperature on both bacterial growth and their capacity to produce protease enzymes. In this study, the isolate *B. stearothermophilus* isolated from olive oil mill sols demonstrated maximum growth at 55 °C (11.05 g/L ± 0.155) and a maximum activity of 6885U/mL ± 91.923 at 55 °C. Similarly, the optimum growth of some *Geobacillus* sp. was attained between 30 °C and 70 °C with the maximum activity at 60 °C [18]. However, it has been reported that the optimum temperature was species-dependent for protease production [19]. Furthermore, the pH of the culture medium is an important criterion for the ability of the microorganism to produce extracellular protease. In fact, the catalytic reaction strongly depends on the charge distribution of substrates and enzyme molecules [20]. It was observed that at acid pH values, the bacterial growth and protease productivity were very low compared with results obtained at pH ranging from 7 to 11. The highest dry weight of biomasses (10.82 g/L ± 0.183) and protease activity (6090 U/mL ± 155.563) were observed at pH 10. Similarly, the majority of investigated *Geobacillus* spp. have their growth at a pH range of 6–9 for the production of protease [21]. However, the maximum growth and protease production by *Bacillus* sp. were achieved at pH 8 [22]. We clearly noticed a relationship between the growth of bacteria and their production of the protease enzyme with regard to temperature and pH optimization. It is well established that carbon and nitrogen sources significantly affect the cell growth and protease production from bacterial strains. The ability of an organism to break down different carbohydrates could be a result of its preference towards living in an environment containing low in organic, and therefore the need to develop its metabolic system to adjust assimilate [23]. In fact, in the present study, galactose permitted the highest protease activity of 8077 U/mL ± 173, followed by sucrose with 2647 U/mL ± 144. Compared with several studies, further carbohydrates were found to increase the production yield of alkaline protease. In fact, Malathi and Chakraborty [24] confirmed that maltose allows the highest protease production yield. Similarly, Tsuchiya et al. [25] reported the use of maltose as a good carbon source for high production of the protease enzyme. Sucrose and fructose have also been described to improve protease production [26]. Glucose is frequently used as a carbon source to produce protease enzymes through fermentation. However, results from different investigations have shown that the production of protease enzyme was reduced by glucose due to glucose catabolic repression [27]. Accordingly, the curves obtained in this study compared with the effect of nitrogen sources showed that the use of yeast extract (1% *w*/*v*) allowed the maximum cell growth (10.525 g/L ± 0.243) and protease production (7170 U/mL ± 113.13). In fact, yeast extract is considered as a good source for the maximum production of protease due to its richness of amino acids, various vitamins, and carbohydrates to improve both the cell growth and metabolic functions of the bacterium [28]. Evidently, the use of 3% yeast extract improved the proteolytic activity and dry weight biomass, which reached 9050 U/mL ± 212 and 11.05 g/L ± 0.12, respectively. This is consistent with the findings of Khusro [29] who reported that yeast extract played a vital role in the production of the enzyme due to the presence of important elements and growth factors required for microorganisms [20]. Several studies have reported that the use of casein 1% (*w*/*v*) significantly affected the production of protease by bacteria [3]. The enhancement of protease production by *B. stearothermophilus* involved studying the effects of incubation time and inoculum size. First, we noticed that protease production was proportional to cell growth, with an optimum production of 5100 U/mL ± 212 being obtained at 24 h. Then, there was a rapid decrease in protease activity and cell growth by up to 72 h at a constant rate. This finding is in agreement with a previous report showing that the production of protease (1900 U/mL) is proportional to the bacterial growth, which was observed at 42 h of incubation [21]. Second, the protease activity was found to be proportional to the inoculum size tested, and the highest productivity (5050 U/mL) was observed with 8 × 10^8^ CFU/mL. After purification of the protease using reversed-phase analytical HPLC eurospher 100, C-8 column (250 × 4.6 mm), SDS-PAGE analysis of the obtained fractions clearly indicated that the protease possesses a molecular mass of approximately 28 kDa. The N-terminal sequence of Ba.St.Pr exhibited a high level of identity of approximately 95% with those of *Bacillus* strains. Alignment of the Ba.St.Pr N-terminal sequence with the highest-identity protease sequences from *Bacillus* strains confirms that the current protease is serine protease. In fact, 95% identity was obtained with alkaline serine protease isolated from *B. altitudinis*, alkaline protease isolated from *B. pumilus*, serine alkaline protease isolated from *B. circulans*, serine protease isolated from *Alkalihalobacillus lehensis*, with the accession numbers APZ77034.1, QJX57841.1, ADN04910.1, and AFP23380.1, respectively. The 5% difference in the aminoacids sequence with other bacillus proteases strongly suggested that the Ba.St.Pr enzyme was a novel serine protease.

The effects of extreme pH values and temperatures strongly argue the use of the pure protease obtained in this study in industrial applications. In fact, Ba.St.Pr exhibited the highest relative activity on casein at pH 10 and 70 °C. Moreover, studies on pH stability at alkaline values demonstrated that at pH 10, Ba.St.Pr remains stable (90%) for 10 h of incubation time. The thermal stability confirmed that Ba.St.Pr is extremely stable as it retained its activity at 80 °C for 4 h when combined with Ca^2+^ and mannitol. The half-life time deduced from the thermal stability curves was approximately 123 min at 80 °C for Ba.St.Pr alone, and it improved to 166 and 187 min when incubated with Ca^2+^ and the combination of Ca^2+^/mannitol, respectively. Compared with proteases derived from *Bacillus* strains, the present study dealt with a thermostable protease. In fact, studies conducted on protease isolated from *B. safensis* reported that the half-life time measured at 70 °C was approximately 90 min in the presence of 2 mM CaCl_2_ [30].

Previous studies have described Ca^2+^ as a stabilizer of the activity and stability of proteases derived from *Bacillus* strains [31]. It has also been reported that polyols prevent the enzyme from denaturation by increasing some hydrophobic interactions within protein molecules [32]. Similarly, some surfactants, oxidizing agents, organic solvents, and ions are used in industrial applications. Thus, it is appreciable to produce resistant enzymes. In the present study, a higher stability of the enzyme was observed in the presence of Triton X-100, Tween 20, or Tween 80, with an increase in its residual activities by 40%, 30%, and 20%, respectively. Moreover, we observed that Ba.St.Pr was remarkably stable in chloroform and ethanol as its activity was improved by 60%, followed by that in DMF, isopropanol, and DMSO in which the residual enzyme activity was 100%. The stability in the organic solvents argue the application of the novel protease in organic synthesis [14]. Furthermore, the enzyme showed very slight loss in its activity after exposure to various commercial detergents, suggesting its potential use as an additive in detergent formulations.

Consequently, all the evaluated activity parameters, including thermotolerance as well as tolerance to a varied range of pH values and stability of enzyme activity over a range of temperatures and pH values, are of interest to the detergent industry for use in the removal of proteinaceous stains. This enzyme can be applied in more than one specific industrial application, such as bioactive peptides used in the food and pharmaceutical industries.

## 4. Materials and Methods

### 4.1. Protease Assay

Enzyme activity was evaluated according to the method described by McDonald and Chen [33]. Briefly, 2 mL of 1% (*w*/*v*) casein in glycine-NaOH buffer (pH 10) was supplemented to 1 mL of enzyme and incubated at 60 °C for 15 min. Then, 3 mL of 10% TCA was added. The control sample contained 2 mL of 1% (*w*/*v*) casein solution, 1 mL of enzyme, and 3 mL 10% TCA (*w*/*v*). Alkaline copper reagent (5 mL) was added to 1 mL of the incubated enzyme/substrate. After 15 min, 0.5 mL of Folin–Ciocalteu reagent was diluted in a 1:1 ratio and left to stand for 30 min. The absorbance was read at 700 nm. One unit of enzyme activity was defined as the amount of enzyme that released 1 µg of tyrosine per mL per min under the above-described assay conditions.

### 4.2. Effects of Culture Conditions on Bacterial Growth and Protease Production of the Isolate B. stearothermophilus

The effects of different parameters on the cell growth and protease production of *B. stearothermophilus* were investigated. The physical and chemical parameters, such as carbon and nitrogen sources, temperature, initial pH, inoculation size, and incubation period of the bacterium were analyzed according to the method described by Sepahy and Jabalameli [22] with few modifications. First, the cell growth was examined by measuring the optical density (OD) at 600 nm. Optimization of the inoculum size ranging from 10^8^ to 8 × 10^8^ CFU/mL under the culture conditions for protease production from *B. stearothermophilus* was examined using the method described by Sinha and Khare [34] at pH 10 in a medium containing 5 g bactopeptone, 5 g yeast extract, 3 g NaCl, 0.2 CaCl_2_, 5 g KH_2_PO_4_, and 0.1 g FeSO_4_·7H_2_O dissolved in 1 L of distilled water. The inoculums were commonly grown in LB broth medium. Protease production assay was conducted as described previously [34] after centrifugation at 23,000× *g* for 15 min at 4 °C. The experiments were performed in triplicate. This method was applied to all the following parameters.

#### 4.2.1. Effect of Inoculum Size

The effect of inoculum size on protease activity was analyzed as follows: 50 mL of the selected fermentation medium was inoculated with various amounts of inoculums ranging from 10^8^ to 8 × 10^8^ CFU/mL (*v*/*v*) bacteria grown overnight. After 48 h of incubation, the protease activity was measured in the obtained culture filtrate. Media were incubated in a shaking incubator at 37 °C for a period of 2 days and assessed for protease activity.

#### 4.2.2. Effects of pH and Temperature:

Briefly, approximately 8 × 10^8^ CFU/mL was inoculated into sterilized LB broth, followed by incubation separately at various pH values ranging from 3 to 12 and various temperatures ranging from 30 °C to 65 °C for 24 h with 200 rpm agitation. Both protease activity and bacterial biomass were evaluated.

#### 4.2.3. Effects of Carbon Sources:

Several media were prepared at pH 10 in 50-mL flasks containing 1% (*w*/*v*) of selected carbon sources. Briefly, casein, xylose, glucose, starch, lactose, glucose, and galactose were tested separately for protease activity and dry weight measurement. The carbon source media were sterilized at 121 °C for 15 min before inoculation with 10% (*v*/*v*) *B. stearothermophilus* and incubated at 55 °C for 24 h with 200 rpm of agitation.

#### 4.2.4. Effects of Nitrogen Sources

As described in the previous section, growth media were prepared containing 1% (*w*/*v*) of the following selected nitrogen sources: ammonium nitrate, ammonium chloride, ammonium dehydrogenate phosphate, tryptone, peptone, or yeast extract. The media were set at pH 10 and sterilized by autoclaving before inoculation with 10% (*v*/*v*) of bacteria and then incubated at 55 °C for 24 h.

#### 4.2.5. Effects of Incubation Time

Microbial growth and enzyme production were investigated at various incubation times in the appropriate optimized medium. Samples were collected at a regular interval of 4 or 8 h. Growth (OD) and protease activity were measured using a spectrophotometer.

### 4.3. Protease Purification Procedure

The isolated protease was purified using a combination of ammonium sulfate fractionation (30–65%) precipitation, heat treatment (80 °C for 30 min), and reversed-phase RP-HPLC (Applied Biosystems, Foster City, CA, USA) on C-8 column. To remove microbial cells, 300 mL of a 24h culture of *B. stearothermophilus* was centrifuged at 9000× *g* for 30 min. The extracellular protease was initially saturated with solid (NH_4_)_2_SO_4_ at 30% after centrifugation at 10,000× *g* for 30 min. The resulting supernatant was saturated up to 65% and recentrifuged and recuperated in 50 mM Tris-HCl buffer containing 2 mM CaCl_2_ at pH 10. The resuspended precipitate obtained after centrifugation at 15,000× *g* for 30 min was subjected to a brief heat treatment step for 30 min at 80 °C, and the denatured material was removed by centrifugation at 10,000× *g* for 30 min. The obtained supernatant exhibiting protease activity was loaded onto a reversed-phase RP-HPLC eurospher 100 (Applied Biosystems, Foster City, CA, USA), C-8 column (250 × 4.6 mm), previously equilibrated with 0.1% TFA in water. Protein elution was performed with an acetonitrile linear gradient (0–80%) at a flow rate of 1 mL/min over 60 min. The sample with the highest protease activity was collected, lyophilized, and stored at 4 °C until further use.

### 4.4. Protein Analysis

The protein content was determined at 595 nm using Bio-Rad DC Protein Assays (Hercules, CA, USA) using the Bradford method [35] with crystalline bovine serum albumin as a standard. The purity and molecular mass of the isolated protease were estimated by sodium dodecyl sulfate polyacrylamide gel electrophoresis (SDS-PAGE) using 15% polyacrylamide gels in the presence of β-mercaptoethanol as a reducing agent [36], and its N-terminal sequence was determined using Edman’s degradation technique as described by Hewick et al. [37].

### 4.5. Amino Acid Sequencing

The bands of purified Ba.St.Pr enzyme were separated on SDS gels and transferred to a ProBlott membrane (Applied Biosystems, Foster City, CA, USA), and the NH_2_-terminal sequence analysis was performed by automated Edman’s degradation using a protein sequencer (Applied Biosystems Protein sequencer ABI Procise 492/610A) equipped with a 140C HPLC system (Applied Biosystems) according to standard operating procedures. The amino acid residues were detected as individual signals.

### 4.6. Effects of pH and Temperature on Ba.St.Pr Activity and Stability

The effects of pH and temperature on the activity and stability of pure protease were analyzed in the presence and absence of calcium ions (3 mM). First, buffers at 200 mM concentration, corresponding to pH values ranging from 2 to 13, were used to test the protease activity at 55 °C. The pH stability of the purified protease was investigated by incubating the enzyme at various pH values ranging from 7 to 13 and at time intervals of 0–36 h. The residual protease activity was determined using the standard assay method after removing the denatured proteins. Measurements were performed in triplicate. Second, the effect of temperature on protease activity was determined by evaluating the enzyme activity at temperatures ranging from 40 °C to 95 °C at pH 10. Finally, to evaluate the thermal stability, pure protease was stored at extreme temperatures ranging from 70 °C to 90 °C, at pH 10, and for time periods ranging from 1 to 10 h. The residual enzyme activity was measured after removing the denatured proteins.

### 4.7. Performance Evaluation of Purified Ba.St.Pr

#### 4.7.1. Effect of Organic Solvents on Enzyme Stability

The organic solvent stabilities of the protease produced by *B. stearothermophilus* were investigated by incubating the enzyme preparation with different organic solvents at 25% (*v*/*v*) for 24 h at 40 °C. The residual proteolytic activities were evaluated at 70 °C and pH 10. The control (100%) used in this study was the reaction mixture without any additives. The following organic solvents were tested: acetonitrile, benzene, chloroform, DMF, DMSO, ethanol, isopropanol, and methanol. A commercial protease (Alcalase 2.5 L, type DX) was used as a positive control.

#### 4.7.2. Effects of Surfactants and Oxidizing Agents on Enzyme Stability

The effects of some neutral and charged surfactants (1%) such as Tween 20, Tween 80, Triton X100, and NATDC on the stability of pure protease were investigated by incubating the enzyme preparation with various surfactants for 24 h at 40 °C under constant shaking at 160 rpm. The residual enzyme activity was assayed at 70 °C and pH 10. The stability of the protease was also evaluated in the presence of oxidizing agents such as H_2_O_2_, sodium perborate, and sodium hypochlorite (1%). The residual enzyme activity was measured under the same conditions and compared with a positive control (Alcalase 2.5 L, type Dx, Novozymes A/S, Copenhagen, Denmark) and a negative control (the reaction mixture without any additives).

#### 4.7.3. Effects of Liquid and Solid Detergent on Enzyme Stability

For evaluating the stability and compatibility of the protease with detergents, liquid and solid commercial detergents were diluted in tap water to obtain a final concentration of 7 mg/mL to simulate washing conditions. The endogenous proteolytic enzymes present in these laundry detergents were denatured by heat treatment of the diluted detergents at 90 °C for 1 h. Next, 15 U/mL of the protease was incubated under shaking with each laundry detergent for 1 h at 40 °C, and the residual enzyme activity determined at pH 10 and 70 °C was compared to that of commercial Alcalase 2.5 L, type Dx.

## Figures and Tables

**Table 1:** Flow sheet for Ba.St.Pr purification (300 mL culture).

Purification Step	Total Activity (Units)	Protein (mg)	Specific Activity (U/mg)	Activity Recovery (%)	Purification Factor
Culture supernatant	2,775,000	3780	734	100	1
(NH_4_)_2_SO_4_ Precipitation (30–65%)	2,358,750	1120	2106	85	2.87
Heat treatment (80 °C for 30 min)	2,090,500	119	19,248	75.3	26.2
RP-HPLC (C-8)	1,534,575	26	59,022	55.3	80.4

One activity unit is defined as the amount of enzyme that hydrolyzed the substrate and produced 1 μg of amino acid equivalent to tyrosine per minute under the experimental conditions used in this study.

## Data Availability

The data presented in this study are available on request from the corresponding author.

## Sample Availability

Samples of the compounds are not available from the authors.
